# Medical Education in Scotland

**Published:** 1893-09-16

**Authors:** 


### MEDICAL EDUCATION IN SCOTLAND.

If Scottish medical schools were frequented only of Scottish students the class rooms at Edinburgh, Glasgow, and Aberdeen would be much less full than they are. It is difficult to ascertain precisely what the proportion of "foreign" to native students at these Universities and schools is; but it is well-known to those who are familiar with the facts that not England only, but all the British colonies contribute their quota to the roll of Scottish undergraduates. Why is this? For two simple reasons. In Scotland there are facilities for obtaining the University degree in medicine which are not to be had in England, and in Scotland living costs less, and education is cheaper. Having said this, one must hasten to add, lest a false impression should be created, that easiness does not in this case imply inferiority. Scottish medical degrees are held in deserved respect the world over. The examinations for the M.B. of Edinburgh are perhaps not so difficult as those for the M.B. of London, and it costs very much less money to become a graduate of Glasgow or Aberdeen than of Oxford or Cambridge; a Scotsman may, however, be pardoned for directing attention to the fact that the student who has taken a full course at one of the London hospitals, unless he is prepared to enter for the degree of the University of London, and has complied with all the conditions to enable him to do so, must be content with the diploma of the Conjoint Board; whereas the student who has taken a course very much the same at a Scottish University, sits for the University degree as a matter of course. Apart from all question as to the relative efficiency of the training, few will venture to deny that the degree of a University, whether of England or Scotland, is of greater value than a bare license to practice. Fees are lower at the Scottish universities, and board and lodgings cost much less than they do in London. The Scottish youth who supported himself at the University in winter on his earnings at farm work in summer, and who lived for the most part on oatmeal, has disappeared into the past; nevertheless his successors contrive to live comfortably, on simple but wholesome fare at an expenditure which amazes those who are accustomed to the more costly and luxurious life of the southern city. It is a fact which may easily be verified that in the city of Aberdeen it is not at all impossible for a student who is willing to share rooms with a companion to obtain good food and respectable board at a cost of no more than ten shillings a week.

The Scottish Universities, which have all medical faculties, are those of Edinburgh, Glasgow, Aberdeen, and St. Andrews with which is affiliated University College, Dundee. The three medical corporate colleges in Scotland are the Royal College of Physicians, Edinburgh, the Royal College of Surgeons, Edinburgh, and the Faculty of Physicians, of Glasgow. These three form the Scottish Conjoint Board.

The fame of Edinburgh in medicine is world-wide. One sometimes hears those who think the past days better than the present speak as if the glory of Edinburgh had departed. They recall the names of physicians, surgeons, and teachers who fifty years ago shed lustre on the Scottish capital, and they ask in dejected tones whom has the Edinburgh of to-day to compare with these. But it is forgotten that the conditions which gave these great men their prominence are no longer present. The field has grown so much larger that individuals, however great, no longer command the attention that they once commanded. The names of the men who do the work of medical teaching in Edinburgh to-day are not so often in the mouths of the public as those of Simpson and Syme and Liston were; nevertheless one may fairly contend that the present Edinburgh staff is as good an all-round staff as ever it was in the palmiest days of the school. Individuals may stand out less, but the general level of knowledge and teaching power is very high. At the same time Edinburgh has suffered some of the penalties of a great reputation. The school is the largest medical school in this country. This having been said, there is no reason why it should not be frankly added that it is too large—that it is overcrowded. In regard to several of the classes this is an advantage rather than a disadvantage, but where clinical work is concerned it makes individual teaching difficult, if not impossible, and it puts the student at a disadvantage in respect of practical work in the wards. The two great teaching bodies in Edinburgh are the University and the School of Medicine. One of the best things in connection with medical education in Edinburgh is that the lectures in the School of Medicine are recognised by the University. Under the recent ordinances one half of the qualifying classes required for graduation at the University may be attended at the school; and it is easy to realise the good effect that the healthy rivalry engendered by this arrangement must have. The School of Medicine has special classes for women which are recognised by the Scottish Conjoint Board. Edinburgh has also its separate school of medicine for women, of which Dr. Sophia Jex-Blake, who was largely instrumental in founding the school, is dean. Students at the University and the School of Medicine receive clinical instruction at the Edinburgh Royal Infirmary, which is one of the finest hospitals in this country. It contains 750 beds, and is thus the second largest hospital in Great Britain. The buildings are of comparatively recent erection, and they are universally admired. The Royal Hospital for Sick Children, the Maternity Hospital, the City Fever Hospital, and the Royal Asylum at Morningside are also open to students.

Glasgow, which has an unending dispute with Liverpool as to which is the second city in the kingdom, is justly proud of its University buildings, which occupy a commanding site above the river Kelvin. Adjoining the University is the Western Infirmary, established in 1874, with 400 beds, in which the great majority of the students receive their clinical instruction. The staff of teachers at Glasgow University is a very strong one. In Queen Margaret's College, quite near the University, special classes are conducted for women, and to ladies who have had their training here the degrees in medicine of the University are open. For clinical instruction lady students go to the Royal Infirmary, the older of the two great hospitals of Glasgow, with 582 beds. There are two extra-mural schools in Glasgow. Anderson's College is within a short distance of the University and the Western Infirmary. The classes are recognised under certain conditions as qualifying for the degrees of Glasgow and Edinburgh Universities. St. Mungo's College is close to and in communication with the old or Royal Infirmary, and students enjoy the excellent clinical opportunities that are there afforded. A full course of instruction under excellent teachers is provided.

The most northerly medical school in Britain is that which is connected with the University of Aberdeen. For Aberdeen it may fairly be claimed that one may in this city obtain an excellent medical training and a University degree to boot at less cost than at any other centre of medical education in the kingdom. The school is not overcrowded. The University buildings at Marischal College are about to undergo extensions that will place the Aberdeen Medical School on an excellent footing in respect of buildings, and of all that ample buildings imply for a medical school. The Aberdeen Royal Infirmary will, before many months are over, have been practically rebuilt. The old buildings are to be given up entirely to administration. New surgical wards were opened last year, and new medical wards are in course of erection. In addition to the Royal Infirmary, the Aberdeen Dispensary, the Sick Children's Hospital, the City Fever Hospital, and the Royal Asylum admit students to the privileges of clinical observation and instruction.

As University College, Dundee, has become affiliated with the University of St. Andrews, the medical schools at Dundee and St. Andrews may be reckoned as forming one school, and it is very probable that before long the medical work of the University will be represented solely at Dundee. At present University College at Dundee, is in a position to give a complete course of instruction for the first three years of medical study. The Royal Infirmary at Dundee contains 250 beds. The University of St. Andrews has a body of examiners in medicine, and although it has not in itself a completely equipped medical staff, it confers after examination the degrees of M.B. and C.M. on students who have fulfilled the conditions laid down by the university as to course of study.

